# Editorial: Exercise and aging with musculoskeletal conditions

**DOI:** 10.3389/fresc.2022.902241

**Published:** 2022-09-13

**Authors:** Alice S. Ryan, Monica C. Serra, Vicki L. Gray

**Affiliations:** ^1^ School of Medicine, University of Maryland, Baltimore, MD, United States; ^2^ School of Medicine, The University of Texas Health Science Center at San Antonio, San Antonio, TX, United States

**Keywords:** aging, muscluloskeletal, physical function, tele-exercise, mechano-transduction

**Editorial on the Research Topic** Exercise and aging with musculoskeletal conditions by Ryan AS, Serra MC and Gra VL. (2022) Front. Rehabilit. Sci. 3: 902241. doi: 10.3389/fresc.2022.902241

This research topic, *Exercise and Aging with Musculoskeletal Conditions,* is a collection of contributions highlighting the effects of aging and chronic conditions on the musculoskeletal system and the effect of which exercise has on mitigating these declines. Exercise has many established musculoskeletal health benefits for people with age-related musculoskeletal conditions (i.e., muscle strength, endurance, balance, and body weight management). Therefore, exercise interventions may be able to reduce the onset and progression of age-related musculoskeletal conditions. It is also necessary to understand the biological mechanisms by which exercise affects the musculoskeletal system.

Gollie et al. (2021) in their article highlight the declines in maximal voluntary force of the knee extensors and physical function in participants with stage 3b and 4 chronic kidney disease not on dialysis. The authors report slower sit-to-stand time and reduced knee extensor strength in men with chronic kidney disease (CKD) compared to Veterans without CKD. Pertinent findings include: (1)The ability to modulate a rapid rate of knee extensor force development early in the contraction (<50 ms) was inversely associated with the performance on the sit-to-stand test only in the CKD group; (2) Both groups demonstrated a direct association between the rate of force development from 100 to 200 ms and the maximal voluntary force; and (3) Rectus femoris muscle quality (grayscale) by ultrasound was inversely associated with rate of force development in participants with CKD.

Strength training with eccentric muscle contractions is one type of exercise program that can mitigate declines in muscle function. The exercise effects of eccentric contractions result in a transient reduction in isometric torque. Zwetsloot et al. (2021) used animal models to test skeletal muscle injury and loss of function induced by eccentric exercise. The authors report declines in peak isometric torque were similar between adult and older mice. In their study of the administration of phytoecdysteroids, which are natural plant steroids, Kwetslott and colleagues demonstrated that 20-hydoxyecdysone (20E) increases skeletal muscle recovery after eccentric contraction-induced damage in adult and old mice.

Mitigating declines in muscle function can involve a variety of training paradigms. Aerobic high-intensity interval training (HIIT) is one such method. Yang et al. (2021) tested a protocol for training middle-aged and aged rats on a forced running wheel-bed. The authors tested the protocol in combination with enalapril treatment. The animals in the HIIT groups doubled endurance time compared to sedentary controls. There was no indication that the addition of enalapril was better than HIIT alone. A recent review Hayes et al. (2021) summarized the literature of the use of HIIT in older (>60 years of age) adults and concluded that HIIT improves muscle function and physical performance which may influence risk for sarcopenia.

Understanding the biological mechanisms that affect recovery from age-related musculoskeletal conditions is needed to recognize the most efficacious rehabilitation protocols to promote long-term independence with aging. Leser et al. (2021) reviewed the mechanisms behind the changes in musculoskeletal function with aging and explored defects in mechano-transduction that impact systemic changes in aging (i.e., senescence, inflammation, oxidative stress, and defects in autophagy). Leser and colleagues present several conceptual models of impaired mechano-transduction and the interactions with microtubule changes, cytoskeletal stiffness, how reduced physical activity and aging contribute to a cycle of frailty, how loading and a shift in loading influence the musculoskeletal system, and the mechano-transduction pathway in youth and in aging. The authors conclude their review with insight into exercise rehabilitation and the benefits on skeletal muscle and bone.

Though exercise has been established as an essential component for treating declines in the musculoskeletal system, access to these programs can be limited for many individuals as a result of disabilities and impairments that limit mobility. Amorese / Ryan, (2022) review tele-exercise in musculoskeletal, cardiorespiratory, and neurological populations and reveal promising results with similar improvements in outcomes compared to in-person interventions. The authors provide a series of comprehensive tables which outline the various tele-exercise programs including the modes of delivery, the duration, frequency and main results for the age-associated diseases. The authors concluded that many studies lacked a long-term follow-up to determine whether the improvements persisted and suggest the need for future investigation. Furthermore, the authors speculate that with advancements in technology, tele-exercise training may be a promising tool to improve health in musculoskeletal disease, neurological disease, and cardiovascular disease in aging.

Together, these articles emphasize recent continued support for the role of exercise in preventing the onset and progression of age-associated declines in musculoskeletal function, while also highlighting some promising potential mechanisms by which exercise may influence this system. They further draw attention to the need for future studies to utilize advancements in technology and enhance our understanding of the mechanisms supporting the benefits of exercise to continue to support the development and evaluation of efficient and effective ways to utilize exercise to promote musculoskeletal health in older adults.

